# Using OPM-MEG in contrasting magnetic environments

**DOI:** 10.1016/j.neuroimage.2022.119084

**Published:** 2022-03-09

**Authors:** Ryan M. Hill, Jasen Devasagayam, Niall Holmes, Elena Boto, Vishal Shah, James Osborne, Kristina Safar, Frank Worcester, Christopher Mariani, Eliot Dawson, David Woolger, Richard Bowtell, Margot J. Taylor, Matthew J. Brookes

**Affiliations:** a Sir Peter Mansfield Imaging Centre, School of Physics and Astronomy, University of Nottingham, University Park, Nottingham NG7 2RD, UK; b Diagnostic Imaging, Neuroscience & Mental Health Programme, The Hospital for Sick Children. 555 University Ave, Toronto, Ontario, Canada; c QuSpin Inc. 331 South 104th Street, Suite 130, Louisville, CO 80027, USA; d Cerca Magnetics Limited, Headcorn Road, Staplehurst, Kent, UK

## Abstract

Magnetoencephalography (MEG) has been revolutionised by optically pumped magnetometers (OPMs). “OPM-MEG ” offers higher sensitivity, better spatial resolution, and lower cost than conventional instrumentation based on superconducting quantum interference devices (SQUIDs). Moreover, because OPMs are small, lightweight, and portable they offer the possibility of lifespan compliance and (with control of background field) motion robustness, dramatically expanding the range of MEG applications. However, OPM-MEG remains nascent technology; it places stringent requirements on magnetic shielding, and whilst a number of viable systems exist, most are custom made and there have been no cross-site investigations showing the reliability of data. In this paper, we undertake the first cross-site OPM-MEG comparison, using near identical commercial systems scanning the same participant. The two sites are deliberately contrasting, with different magnetic environments: a “green field ” campus university site with an OPM-optimised shielded room (low interference) and a city centre hospital site with a “standard ” (non-optimised) MSR (higher interference). We show that despite a 20-fold difference in background field, and a 30-fold difference in low frequency interference, using dynamic field control and software-based suppression of interference we can generate comparable noise floors at both sites. In human data recorded during a visuo-motor task and a face processing paradigm, we were able to generate similar data, with source localisation showing that brain regions could be pinpointed with just ~10 mm spatial discrepancy and temporal correlations of > 80%. Overall, our study demonstrates that, with appropriate field control, OPM-MEG systems can be sited even in city centre hospital locations. The methods presented pave the way for wider deployment of OPM-MEG.

## 1. Introduction

Magnetoencephalography (MEG) measures magnetic fields around the head generated by neural current flow (Cohen, 1972). Mathematical modelling of these fields enables generation of 3D images, showing the moment-to-moment evolution of electrophysiological brain activity (Baillet, 2017; Hämäläinen et al., 1993). The fields generated by the brain are small (~10^−13^ T) and to gain sufficient sensitivity, conventional MEG scanners use superconducting quantum interference devices (SQUIDs) which must be cryogenically cooled to liquid helium temperatures (Jaklevic et al., 1964). This places significant limitations on the utility and practicality of the available instrumentation. However, MEG system design has been revolutionised by the availability of small, lightweight, and robust optically pumped magnetometers (OPMs) (Alem et al., 2014, 2017; Allred et al., 2002; Borna et al., 2017; Boto et al., 2017; Kominis et al., 2003; Schwindt et al., 2007). OPMs exploit the quantum properties of alkali atoms to measure local magnetic field with high precision. Sensitivity is approaching that of a SQUID, and because the sensors do not require cryogenics, they can be placed closer to the scalp surface, improving sensitivity, spatial resolution, and the uniformity of coverage (Boto et al., 2016; Hill et al., 2020; Iivanainen et al., 2017). Flexible placement of sensors also allows for lifespan compliance (Hill et al., 2019), and assuming background fields are appropriately controlled (Holmes et al., 2018), subjects can move during a scan (Boto et al., 2018). In this way, OPMs are opening new avenues for MEG research, enabling novel experimental design, new subject cohorts, and better data. This, coupled with lower purchase and running costs, makes OPMs arguably the most attractive building block for future generations of MEG instrumentation (although we note other technologies also offer significant promise (Schneiderman, 2014; Webb et al., 2020)).

Despite the promise, significant hurdles remain for OPMs to overtake SQUIDs as the MEG sensor of choice. Perhaps the biggest barrier relates to the magnetic environment in which systems are housed. Magnetic fields from the brain are much smaller than the fields that exist naturally in the environment. For this reason, MEG systems are usually operated inside a magnetically shielded room (MSR), formed from separate layers of high permeability and high conductivity metals (usually mu-metal and aluminium). These act to reduce low frequency, and high frequency interference fields, respectively. However, the requirements for shielding for an OPM system are even more stringent than for SQUID systems; there are three reasons for this. First, OPMs are “zero-field ” magnetometers, meaning that their operation is reliant on the background temporally stationary (henceforth termed “static ”) magnetic field being close to zero (in practice this field can be controlled by “on-board ” electromagnetic coils, but the starting field must still be < 50 nT). This is distinct from SQUIDs which are relatively unaffected by static magnetic fields. In most magnetically shielded rooms fields, although static field is reduced by flux-shunting in mu-metal walls, the presence of the mu-metal itself leaves a remnant field inside the room, which can be greater than the operational level of an OPM. Second, once in operation, OPMs have a low dynamic range. This is because as field is increased, the linearity of the OPM response to field is lost^1^; a change in background field of ~3.5 nT would be equivalent to a gain error of 5% (www.quspin.com), raising to 10% for a field change of 5 nT. This means that if the field drifts over time (e.g., due to environmental changes), or equivalently the OPM array moves with respect to a temporally static field (which also causes a change in field), the OPM measurement will be compromised, and the data quality impacted. Consequently, both low frequency environmental drifts and static field must remain at a level of < 3.5 nT (i.e., within 5% gain error) throughout an experiment for effective OPM-MEG operation. Third, as in conventional MEG, magnetic interference from the environment degrades signal-to-noise ratio (SNR). However, most OPMs are formulated as magnetometers whereas flux transformers used for conventional MEG are often gradiometers. Magnetometers are more susceptible to magnetic fields from distant sources and so OPM-MEG is ostensibly more susceptible to environmental interference. In sum, the success of OPM-MEG is dependent on extremely accurate control of background fields. This provides a significant challenge, particularly when siting OPM-MEG systems in regions of high magnetic interference (e.g., city centre sites).

In addition to background field, several other challenges exist; for example, minimisation of crosstalk between sensors, optimised array design, robust sensor mounting, accurate measurement of sensor location and orientation, and adequate thermal regulation to dissipate heat generated by the sensors (to ensure subject comfort) are all requirements for effective OPM-MEG operation. Multiple solutions have been proposed, and a number of effective OPM-MEG arrays are in existence (Borna et al., 2020; Hill et al., 2020; Sander et al., 2020; Seymour et al., 2021). However, the extent to which one can achieve comparable data from multiple sites – particularly if those sites have different levels of magnetic interference – is unclear. The ultimate success of OPM-MEG will require such cross-site robustness. This, coupled with ease of system use and diminished reliance on an extensive (physics-based) support network, is critical if OPM-MEG is to achieve its full potential and ultimately replace SQUID-based MEG systems.

In this paper, we report the first cross-site OPM-MEG comparison. Specifically, we contrast identical OPM-MEG arrays in very different magnetic environments. The first is a “green field ” (campus university) site with an OPM-optimised magnetically shielded room; the second is a city centre hospital site with OPM-MEG installed in an existing (non-optimised) magnetically shielded room. In what follows, we first demonstrate that by a combination of hardware (Holmes et al., 2019) and software (Tierney et al., 2021) approaches for interference reduction, OPMs can be made to work with a similar noise floor in both locations. Following this, at both sites, we capture OPM-MEG data during both a visuo-motor task (well known to generate robust neural oscillatory effects in the beta and gamma bands), and a visual face processing task (known to generate evoked responses from both primary and lateral visual areas) in the same participant. Results from both sites are compared quantitatively, at the sensor level and following source reconstruction.

## 2. Methods

All data were collected by the authors. All code for analysis was custom written by the authors using MATLAB unless otherwise stated.

### 2.1. Site and system descriptions

Our first OPM-MEG system was at the Sir Peter Mansfield Imaging Centre, University of Nottingham, UK (SPMIC) – a site with inherently low magnetic interference. The system was housed inside a magnetically shielded room (Magnetic Shields Limited, Kent, UK) comprising 4 layers of mu-metal and a single layer of copper. Static magnetic field inside the room is minimised by a degaussing system (Altarev et al., 2015) which allows demagnetisation of the inner mu-metal walls. Background static field impinging on the array was expected to be ~2 nT, with low frequency (i.e., < 1 Hz) drifts in magnetic field of ~0.3 nT, measured over a ten-minute recording (Rea et al., 2021).

Our second site was at the Hospital for Sick Children, Toronto, Canada (SickKids). This is a city centre hospital site with high inherent magnetic interference generated by nearby infrastructure including elevators, a metro-line, parking garages and local construction. The SickKids OPM-MEG system was housed in a MSR (Vacuumschmelze, Hanau, Germany) comprising two layers of mu-metal and a single layer of aluminium (this MSR was previously used for SQUID-MEG). No degaussing was available. The static background magnetic field was expected to be ~30–70 nT (~20 times more than the SPMIC site) and maximum field drifts measured over a 10-minute period were expected to be 5–10 nT (~30 times more than SPMIC).

At both sites, the OPM-MEG device was equivalent (Cerca Magnetics Limited, Kent, UK; (Hill et al., 2020)). The array contained 24 dual-axis zero-field magnetometers manufactured by QuSpin Inc. (Colorado, USA). Each sensor is a self-contained unit, of dimensions 12.4 × 16.6 × 24.4 mm 3, containing a Rb-87 gas vapour within a glass cell, a laser for optical pumping, and on-board electromagnetic coils for controlling local magnetic field within the cell. Optical pumping polarises the atomic magnetic moments of the atoms in the gas, inducing a bulk magnetisation. In the presence of an external field (i.e., the neuromagnetic field) this magnetisation obeys the Bloch equations and can be exploited to generate a sensitive measure of local field. Two orthogonal components of the local magnetic field (perpendicular to the pumping laser beam) were measured at each OPM *sensor*, giving a 48-*channel* system (note that the OPMs themselves were oriented so field was measured radial to the head, as well as in one tangential orientation). Each channel had an inherent noise floor (environmental interference notwithstanding) of 7 – 10 fT/sqrt(Hz) and a bandwidth of 0 – ~130 Hz. Analogue signals representing the time evolution of measured magnetic fields were fed from the OPM electronics to a National Instruments digital acquisition system (DAQ), via which they were recorded.

**Fig. 1.**
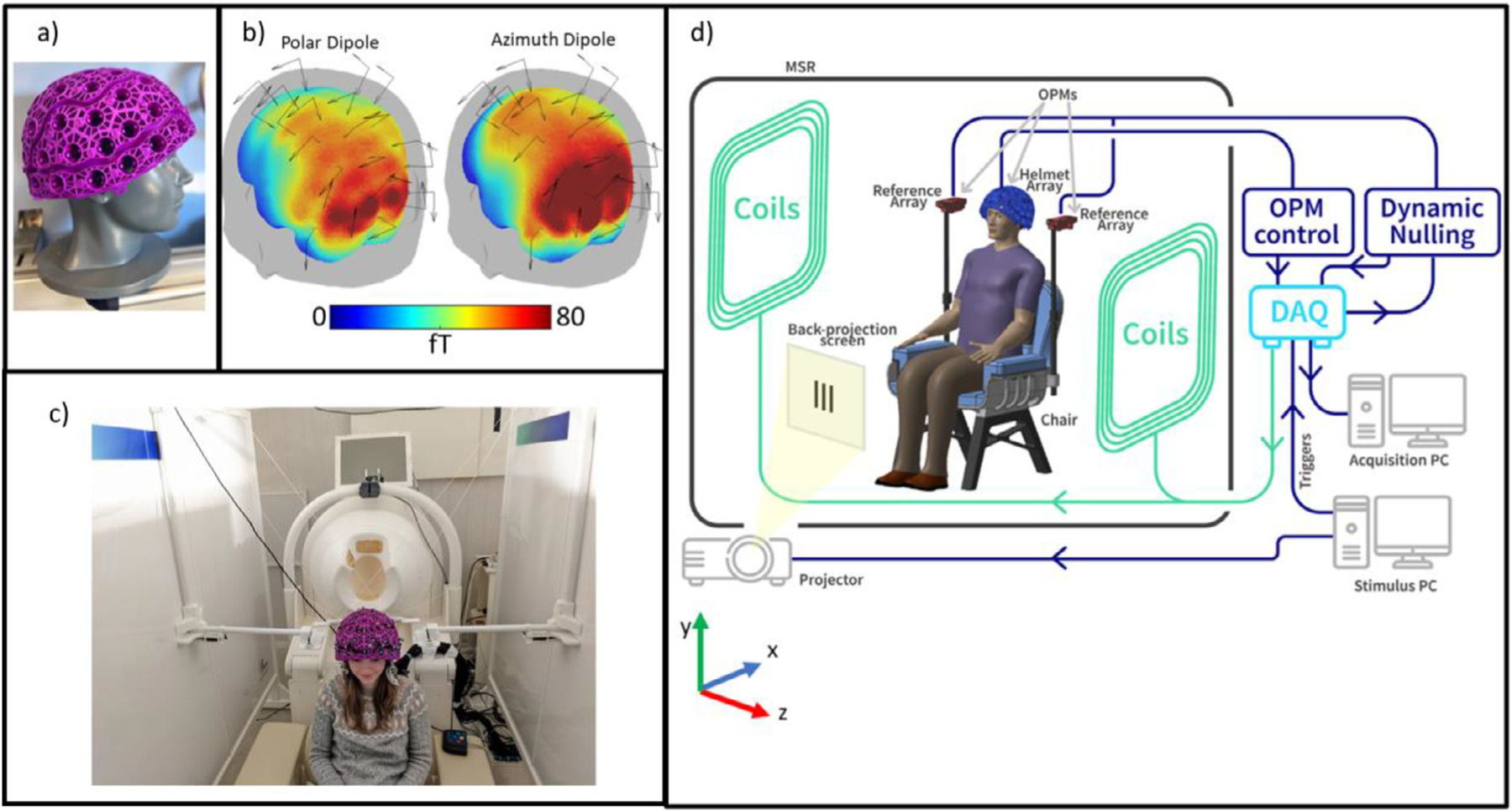
System schematics. a) A lightweight generic helmet designed to fit ~95% of adults. b) OPM placement relative to the head. The coloured surface represents sensitivity to a dipole oriented in the polar (left) or azimuth (right) orientation; we ignore radial dipoles due to the relative insensitivity of MEG to dipoles in this orientation. c) Biplanar coils placed either side of the subject. d) schematic diagram of the Cerca Magnetics OPM-MEG system used at the two sites.

Sensors were mounted on the head via a 3D printed helmet (Cerca Magnetics Limited, Kent, UK – Fig. 1a). The helmet is made from a lattice which makes it lightweight (700 g) and enables heat to escape from the OPMs (which are heated to an external surface temperature of ~≤ 40 °C). The lattice also enables free flow of air to the subject’s scalp and contains features for cable management. The helmet contained 64 possible slots for sensor mounting, and the 24 OPMs used were positioned to cover the left parietal and occipital cortices Fig. 1.b shows a digital representation of the sensor locations with respect to the brain; the arrows represent the sensitive axes along which field was measured. The coloured brain surface represents relative sensitivity to dipoles in different regions. The left-hand figure shows the array sensitivity to dipoles with a polar (Θ) orientation, and the right-hand figure shows the array sensitivity to dipoles with an azimuthal orientation (Φ). The colour represents the Frobenius norm of the lead field from each dipole.

Magnetic field surrounding the OPM helmet was controlled using a set of bi-planar coils placed either side of the participant (Holmes et al., 2018, 2019; Cerca Magnetics Limited, Kent, UK – Fig. 1c). These coils, which are wound on two 1.6-m square planes separated by a 1.5-m gap, generate 3 orthogonal magnetic fields and all 5 independent first-order (i.e., linear) gradients within a 40-cm cube inside which the participant’s head is positioned. A reference array, placed behind the participant, measures the background field/gradient and currents are applied to the bi-planar coils to control this remnant field. At SPMIC, this system of coils was used to remove both the static field and field drift, while at SickKids, only the field drifts were cancelled (described below). Consequently, there is a larger static field at the SickKids site, and so the subject was instructed to sit still during acquisition at both sites (see also Discussion). Fig. 1.d shows a schematic diagram of the complete system. Note in addition to the helmet, coils and MSR, a stimulus delivery system was available in both labs to deliver visual stimuli to the participant via back projection through a waveguide in the MSR and onto a screen placed in front of the subject.

### 2.2. Interference rejection methods

As outlined above, the SickKids site was significantly more challenging than the SPMIC site in terms of background magnetic interference. The expected large drifts in background field regularly caused the OPMs to exceed their operational range (± 3.5 nT). Further, we expected the interference inside the room to be significantly worse than that commonly experienced in SPMIC. For this reason, two separate techniques were used to control interference (at both sites).

- *Dynamic nulling*: To keep the sensors within their operational range of ± 3.5 nT, the bi-planar coils either side of the participant were operated in a dynamic proportional-integrative (PI) mode. A complete description of this has been given elsewhere and will not be repeated here (Holmes et al., 2019; Rea et al., 2021). Briefly, a reference array (Fig. 1d) consisting of four QuSpin OPMs (two placed either side of the subject’s head, separated by ~30 cm), measured the x, y, and z components of the background field at two locations, as well as the field gradients in the z-direction (i.e., dBx/dz, dBy/dz and dBz/dz). The reference magnetometer signals were outputted to a high-speed (60 Hz) PI controller implemented in LabVIEW, which calculates compensation currents which are fed back to the coils. These, in turn, generate temporally changing fields that dynamically compensate < 3 Hz changes in the local magnetic field. In this way, we could control the drifts inside the MSR.
- *Homogeneous field correction (HFC)*: To reduce environmental interference after dynamic nulling, we used Homogeneous Field Correction (Tierney et al., 2021). Briefly, the magnetic interference from distant sources, observed by an OPM array distributed over a relatively small volume (i.e., around the head), can be modelled as a spatially homogeneous magnetic field (i.e., we presume that sources of interference are sufficiently distal that the spatial variation of magnetic field over the head volume is negligible). Assuming the array is distributed appropriately to sample magnetic fields along all three orthogonal axes (which was the case in the present study) then the homogenous field can be estimated. Then, through knowledge of the individual sensor orientations, its manifestation at each sensor can be estimated and subtracted from the data. This acts to reduce external interference and improve signal-to-noise ratio. The low rank of the model (i.e., the assumption of field homogeneity) means that there is little risk of removing substantial neural signal, which has marked spatial variation across the array.

When these two techniques are used in conjunction, we expected the dynamic nulling to control low frequency drifts, and HFC to compensate for interference across the full range of frequencies.

### 2.3. Data collection

To test the effects of interference rejection, 5 min of empty room data were recorded at each site, with and without dynamic nulling. The data with dynamic nulling were further processed using homogeneous field correction. In all three cases (i.e., no correction, dynamic nulling, and dynamic nulling + HFC) the noise floor was assessed quantitatively. The power spectral density within each window was computed using the Matlab (Mathworks Inc.) ‘periodogram’ function with a flattop window, over a frequency range 0 to 100 Hz (resolution of 0.1 Hz).

Following empty room recordings, we acquired human MEG data in a single subject. The participant was a male, aged 26, and right-handed. We performed two experimental paradigms, both well known to produce robust neuromagnetic effects. The first task was a *visuo-motor* paradigm (Hoogenboom et al., 2006; Iivanainen et al., 2020). Each trial comprised 1 s of baseline measurement followed by visual stimulation in the form of a centrally presented, inwardly moving, maximum-contrast circular grating. The grating subtended a visual angle of 7.6° at both sites, and was displayed for a jittered duration of either 2.1 s, 2.2 s or 2.4 s. Each trial ended with a 3-s baseline period, and a total of 100 trials was used. During baseline periods, a fixation dot was shown on the centre of the screen. The participant was instructed to perform abductions of their right index finger for the duration that the stimulus was on the screen to ‘activate’ primary motor cortex. We expected to measure simultaneous fluctuations of beta oscillations in motor cortex, and gamma oscillations in visual cortex. The second task was a *face processing* paradigm. Here, the participant was asked to passively view a series of images, each containing a face. In a single trial, a face was displayed on a screen for 300 ms; this was followed by a rest period of jittered duration (1900 ± 181 ms) during which a fixation cross was shown. A total of 100 trials was recorded. This task is well known to generate robust evoked responses both in primary visual cortex (at a latency of ~100 ms) as well as the fusiform area (at a latency of ~170 ms) (Bentin et al., 1996; Halgren, 2000; Taylor et al., 2001). For both paradigms the subject was seated and free to move but was asked to remain still. MEG data were acquired at a sample rate of 1200 Hz. Each paradigm was independently run 5 times in the same subject at each of the two locations (SPMIC and SickKids). The participant gave written informed consent, and the study was approved by the local research ethics board at both sites.

Following data collection, a 3D optical camera was used to generate a digital model of the location of the helmet (and thus the sensors) relative to the brain anatomy (Hill et al., 2020). A digitisation of the participant wearing the helmet was acquired using a Structure Core 3D scanner (Occipital Inc., San Francisco, CA, USA). This was followed by a second digitisation with the helmet removed and the participant’s hair tied back (to smooth the digitisation of the top of the head). Finally, a structural MRI of the participant’s head was acquired (using a 3T Phillips Ingenia MRI scanner running an MPRAGE sequence with a spatial resolution of 1 mm). An electronic Computer-Aided Design (CAD) file of the helmet with the exact locations and orientations of the sensors was aligned to the first digitisation (of the helmet relative to the face) using 9 identifiable reference points on the helmet. The first digitisation was then aligned to the second using identifiable facial features (e.g., the nasion, the alar facial groove either side of the nose, cheek bones) and an iterative closest point (ICP) algorithm used to fine-tune this alignment (implemented in MeshLab (Cignoni et al., 2008)). The second digitisation was then aligned (using the same method) to the head/face surface extracted from the MRI. This procedure allowed a complete co-registration of the sensor locations and orientations to the anatomical MRI. This would be used later for modelling source locations.

### 2.4. Data analysis

For each recording, following homogeneous field correction, data were bandpass filtered (between 1 and 150 Hz for the visuo-motor paradigm, and 2 and 40 Hz for the face processing paradigm). Bad trials, defined as those in which the standard deviation of the signal at any one sensor was greater than 3 times the average standard deviation of the signal at that sensor across all trials, were removed. Visual inspection of the data confirmed this simple algorithm was successful at removing trials with excessive noise. Following this, we analysed data first in sensor space, and then via source modelling:

#### 2.4.1 Sensor space visualisation

For the *visuo-motor task*, data were further filtered into the beta (13 – 30 Hz) and gamma (35 – 60 Hz) bands. A Hilbert transform was applied to these filtered data, with the absolute value of the resulting analytic signal being used to generate an amplitude envelope (Hilbert envelope) showing modulation of oscillatory amplitude in each band. The envelope was averaged across trials and baseline corrected (the baseline was calculated over the −3.4 *s* < *t* < −2.5 s time window, relative to stimulus offset at *t* = 0 s). The average envelopes for all 5 experimental runs at each of the two sites were then averaged and the standard deviation between runs was found to assess repeatability. This procedure was run for every channel.

To assess sensor space field topography of the beta and gamma band signals, we computed signal to noise ratio (SNR) at each channel. The trial averaged envelope was divided into an “On ” window (i.e., when the stimulation was on; −2 *s* < *t* < −0.5 s) and an “Off” window (i.e., when the stimulus was off; 0.5 *s* < *t* < 2 s). The SNR in the gamma-band was calculated as the difference in mean signal between the windows, divided by the standard deviation of the signal in the Off window. Similarly in the beta-band, the SNR was calculated as the difference in signal means between the two windows, divided by the standard deviation in the On window (note this was to avoid misrepresentation of SNR due to the beta rebound; note also, since the beta amplitude was expected to decrease during stimulation, beta band SNR was expected to be negative). The resulting SNR values were plotted as a flattened topographical map, across sensor locations, to visualise the sensor-space topography of the beta and gamma-band responses. Two separate topographies were derived, one for the radially oriented field, and one for the tangentially oriented field. A time-frequency spectrum (TFS), alongside averaged envelopes for beta and gamma bands, were also constructed for the channels with the highest SNR. The TFS was derived by sequentially filtering signals into overlapping bands, computing the envelope of oscillatory power, averaging over trials, and concatenating in the frequency dimension.

For the face processing task, trials were averaged and baseline corrected (with baseline calculated in the 1 *s* < *t* < 2 s time window; *t* = 0 s corresponds to onset of the face stimulus). The trial-average response for all 5 runs at each site were averaged and the standard deviation found to assess repeatability. The “best ” sensor (i.e., the sensor showing the largest response) was assessed by measuring the range of the trial-averaged signal in the 0.1 *s* < *t* < 0.2 s window. A field map was produced showing the field topography at the time of the largest peak in the evoked response. Again, field maps were made for radially and tangentially oriented fields.

#### 2.4.2 Source modelling

For both paradigms, source modelling was performed using a vector beamformer (Robinson and Vrba, 1998; van Veen et al., 1997; van Veen and Buckley, 1988). The brain was divided into 2-mm cubic voxels, and at each voxel location, beamformer reconstructed source estimates were made for sources in the polar and azimuth orientations. To generate a visualisation of task induced signal modulation across the brain, a pseudo-t-statistical approach was used to contrast source power in active and control windows. For both tasks, the forward solution was calculated assuming a dipolar source, and a single-shell uniform volume conductor head model (Nolte, 2003) created using FieldTrip (Oostenveld et al., 2011).

For the visuo-motor task, the active and control windows were −1.5 *s* < *t* < −0.5 s (i.e., the period where the finger was moving and the visual stimulus was on the screen) and 0.5 *s* < *t* < 1.5 s (i.e., a period where movement had ended, no stimulus was present, and where we expect to observe the post-movement beta rebound (Jurkiewicz et al., 2006; Pfurtscheller et al., 1996)) respectively. All timings are relative to stimulus offset. Images showing the spatial signature of modulation in oscillatory power were generated for both the beta and gamma bands. Beamformer weights were calculated independently for each band, with the covariance matrices generated using a time window spanning the entire experiment (Brookes et al., 2008). The covariance matrices were regularised using the Tikhonov method with a regularisation parameter equal to 5% of the maximum eigenvalue of the unregularized matrix. Based on the pseudo-t-statistical images, a peak location showing maximum oscillatory power modulation was determined, and a signal from this location extracted, again using a beamformer. Here, data covariance was calculated in the 1–150 Hz band and beamformer weights were used to generate a ‘virtual sensor’ time course. Note that a single dipole orientation was chosen to maximise the signal to noise ratio at that location. A time-frequency spectrum was then constructed and averaged over all 5 runs for each site.

For the face processing task, the active and control windows were 0.075 *s* < *t* < 0.175 s (spanning the expected evoked responses in visual and fusiform regions) and 1.075 *s* < *t* < 1.175 s (capturing a rest period) respectively. Timings relative to stimulus onset. Pseudo-T statistical images showing the spatial signature of modulation in task evoked power were generated. The covariance matrix was generated using data filtered in the 2 – 40 Hz and a time window spanning the entire experiment. Again 5% regularisation was used. Two dipole locations were selected – one in the primary visual cortex (MNI coordinates: (−8, −100, 7) mm), and the other at the peak of the average T-stat for each site (in the left fusiform gyrus for both sites; MNI coordinates: (−45, −60, −10) mm) – and a signal in each location was reconstructed using the beamformer. Evoked responses were generated by averaging over trials. These responses were then averaged across all 5 runs for each of the two experimental sites, and a standard deviation calculated to assess robustness. Again, a single dipole orientation was chosen to maximise the signal to noise ratio at that location.

## 3. Results

### 3.1. Rejection of interference

**Fig. 2.**
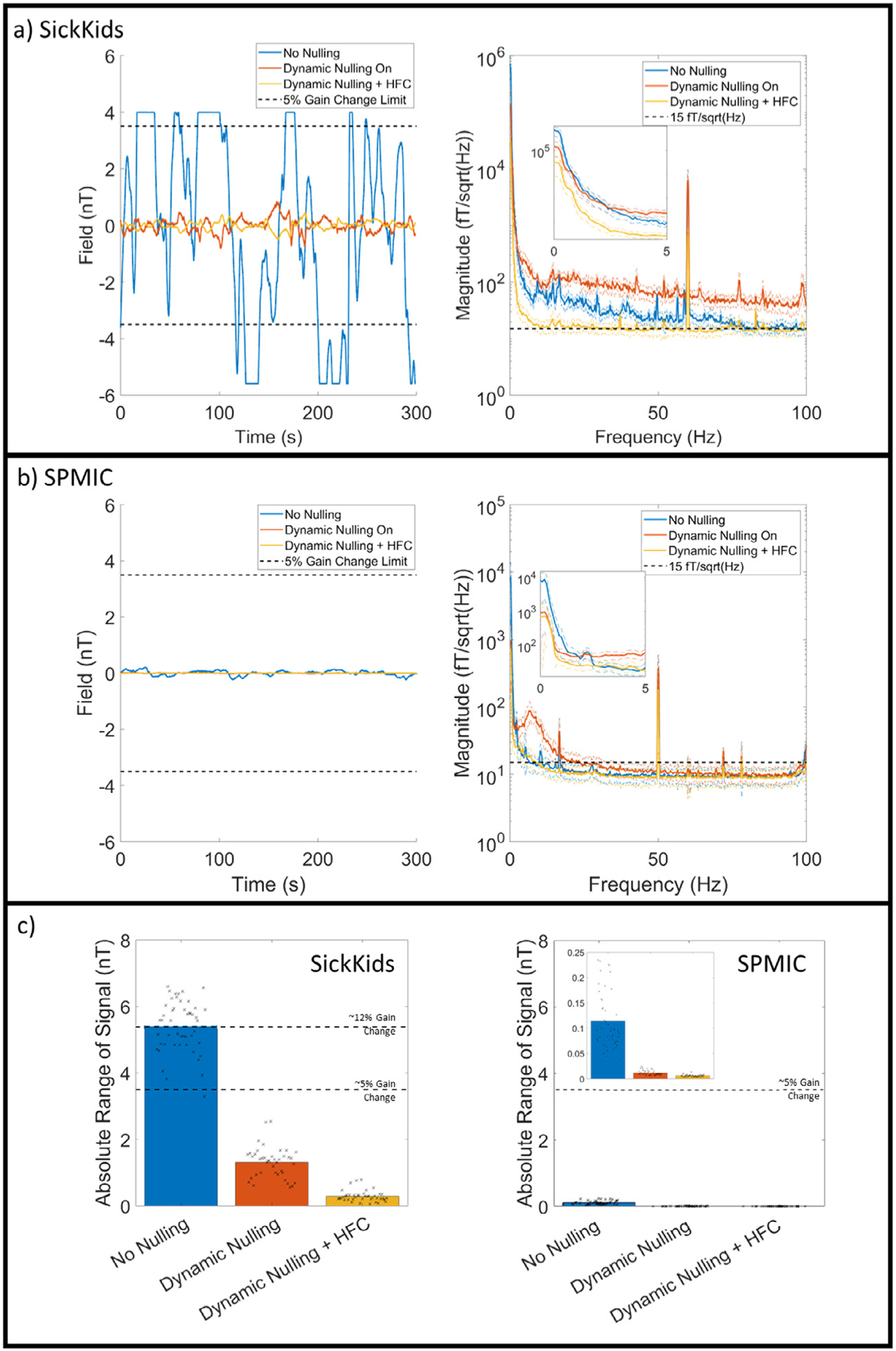
Interference rejection. a) SickKids empty room recording. Raw data for a single representative sensor are shown on the left for the No Nulling Recording (blue), Dynamic Nulling Recording (red), and the Dynamic Nulling Recording with Homogenous Field Correction (HFC) applied (yellow). A dashed line at 3.5 nT represents a gain change in the signal of 5%; if field increases above this line the sensor is non-operational. On the right, the power spectral density (PSD) of each recording is shown, with the inset showing the differences at low (< 5 Hz) frequencies. b) Identical to a) for the SPMIC site. c) Left: the average absolute range (i.e., the largest change from zero) for each recording for the SickKids site. The black dashed lines show the 5% and 12% gain change limits. In both plots, the black crosses show the values for each channel, and the bar the average across all channels. Right: The same plot for the SPMIC site. The inset image shows the same data with the y-axis rescaled.

Fig. 2a and 2b (left panels) shows magnetic field measured over time for a representative sensor placed in an empty helmet at the central region of the bi-planar coils. The recording lasted 5 min. The two black dashed lines at ± 3.5 nT represent a field change corresponding to a 5% change in sensor gain (we would deem sensors inoperable at fields outside of this range). The right-hand panels of Fig. 2a and 2b shows the mean power spectral density over all 24 sensors placed in the helmet, with the inset axes showing data at frequencies < 5 Hz. Here, the black dashed line is at 15 fT/sqrt(Hz); in the absence of external interference (i.e., considering only noise inherent to the sensors) we would expect the power spectral density to be below this line at frequencies above ~5 Hz (most OPMs have inherent noise of ~7 – 10 fT/sqrt(Hz)). For this reason, we deem 15 fT/sqrt(Hz) as the ‘target’ baseline noise level (at which inherent sensor noise dominates environmental interference). In the plots, the blue lines show raw data (i.e., with no dynamic nulling or mean field correction); the red lines show data with dynamic nulling, and the yellow lines show data with both dynamic nulling and homogenous field correction (HFC) applied Fig. 2.a shows the case for SickKids; Fig. 2b shows the case for SPMIC.

At the SickKids site, when no nulling is applied, the background field drifts cause the sensor to regularly exceed its operational range. When dynamic nulling is applied, the sensor is kept within its operational range, but the noise floor above 3 Hz is raised. HFC removes the majority of the interference, bringing the noise floor close to 15 fT/sqrt(Hz). At the SPMIC site, even with no nulling the sensor is well within its operational range. Dynamic nulling reduces the amplitude of the low frequency interference but again increases interference above 3 Hz. HFC again corrects the noise floor above 3 Hz to a level similar to the no nulling case.

These example results are formalised in Fig. 2c. Here, the left panel shows the absolute range (i.e., the absolute value of the maximum change from zero) for all 48 channels in the SickKids array. The black crosses represent the individual values for each channel, while the bar represents the mean across all channels. In the no nulling case, the average range is in excess of 5 nT, which corresponds to a gain change in excess of > 10%, and all but one channel exceed their operational range at some point during the 5-minute recording. However, when dynamic nulling is applied, all channels remain within their operational range, and HFC reduces this further (though it should be noted that this is post-processing so has no effect on gain error). The right panel shows the equivalent data for the SPMIC site (note the difference in the y-axis scale).

These data show clearly that dynamic nulling can be used to maintain sensor operation, even at a site where there are large changes in background field. However, this comes at the cost of increases in higher frequency interference which is generated by noise in the coil current drivers. Consequently, with only dynamic nulling, the background noise is above the 15 fT/sqrt(Hz) target. However, HFC corrects this, as well as supressing other background interference.

In addition to the absolute values of field shown in the Figure, we also measured the standard deviation of the signals expressed as a fraction of the standard deviation of the no-nulling case. (i.e., we took the standard deviation of the signals in the no-nulling case, and used this to normalise all three measurements, and then measured the normalised standard deviations of data with dynamic nulling, and dynamic nulling plus HFC). At the SickKids site, dynamic nulling alone reduced the standard deviation to 18% ± 14% of the uncorrected data. Dynamic nulling plus HFC reduced this further to 4.0 ± 2.8% (values show mean and standard deviation across sensors). At the SPMIC site, dynamic nulling alone reduced the standard deviation to 8.8 ± 3.7%; the addition of HFC further reduced it to 5.4 ± 4.3%. These results confirm quantitatively what is shown in Fig. 2.

### 3.2. Data rejection

In the human experiments, we rejected trials with high levels of interference. These data, for both sites, are shown in Table 1. At the SickKids site, on average 22% of the trials for the visuo-motor task had to be discarded (~2 min of data) due to interference; likewise, 20% of the trials were discarded for the face processing task. At the Nottingham site, these values were reduced for the face processing task (5.6% of trials), while only slightly for the visuo-motor task (16% of trials). These data will be further discussed in Section 4.

### 3.3. Visuo-motor task results

**Fig. 3.**
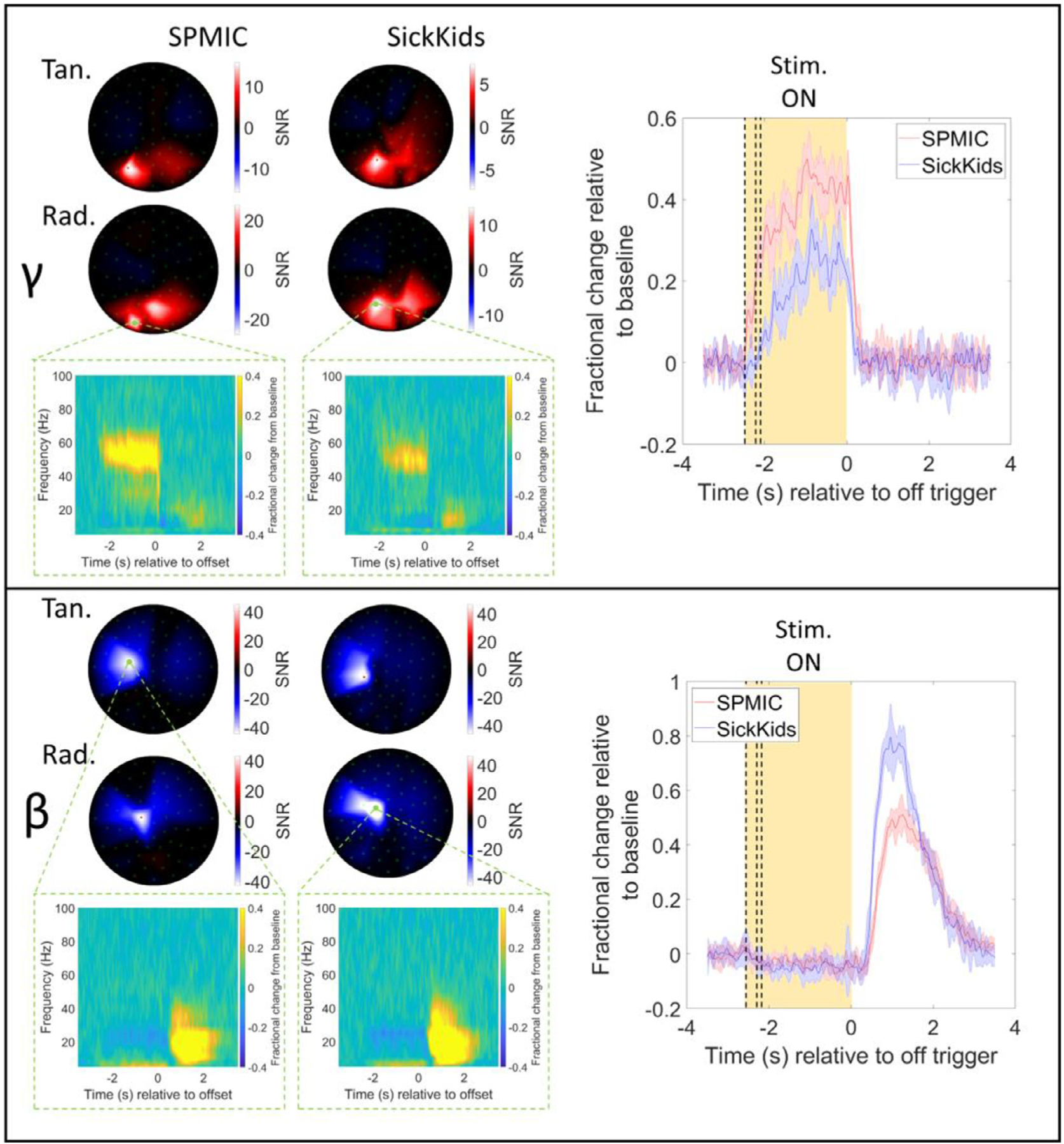
Visuo-motor results (sensor-level). Upper panel: Sensor-level results for the gamma-band (35 – 60 Hz). Spatial topography of the signal-to-noise ratios (SNR) for each sensor averaged across all 5 runs is shown for each site (tangential-axis measurements on top, radial-axis on bottom). On the right, the trial-averaged envelope for the sensor with the highest SNR in the beta band is plotted, with shaded error bars showing the standard deviation across all 5 runs. The yellow shaded region shows the active window, with dashed lines showing the jittered durations of 2.1, 2.2, and 2.4 s. Results for each site are overlaid, SPMIC in red and SickKids in blue. Time-frequency spectrograms are also shown for the sensor with the highest SNR. Lower panel: Same as the upper panel but in the beta-band (13 – 30 Hz).

Fig. 3 shows sensor-space betaand gamma-band signals recorded during the visuo-motor task. The spatial topographies show average (across all 5 runs) SNR for each sensor, for each frequency band and experimental site. The line plots show the trial-averaged oscillatory envelopes from the sensor with the largest SNR, averaged over the 5 runs with the standard deviation represented by the shaded areas. A time-frequency spectrum for the largest SNR sensor is also shown. Note that for the beta band, the SNR at the ‘best’ channel was 53 at SickKids and 45 at SPMIC. for the gamma band, the SNR was 7 at SickKids and 16 at SPMIC

In the occipital sensors we observe gamma synchronisation during visual stimulation (in the −2 *s* < *t* < 0 s window). Meanwhile, in the sensors over the motor cortex, we observe the characteristic beta-band desynchronisation (during the − 2 *s* < *t* < 0 s window), followed by the post-movement beta rebound (during the 0 *s* < *t* < 2 s window). The beta rebound amplitude is slightly higher for the SickKids site compared to the SPMIC. Whilst the reason for this is unknown, it is likely to be a result of placement of the sensors. In contrast, the gamma signal is higher in amplitude at the SPMIC site. Again, this could be due to the way in which the helmet was positioned, or it could result from differences in either the way the visual stimulus was either presented/viewed or indeed the state of the subject at the time of the experiment. Note that this difference in fractional change is largely responsible for the SNR changes that are observed in the topographical plots. Note there is an apparent frequency shift in gamma at the two sites, however this is an artefact caused by masking of the gamma signal due to powerline noise (the frequency of which differs across the 2 sites).

**Fig. 4.**
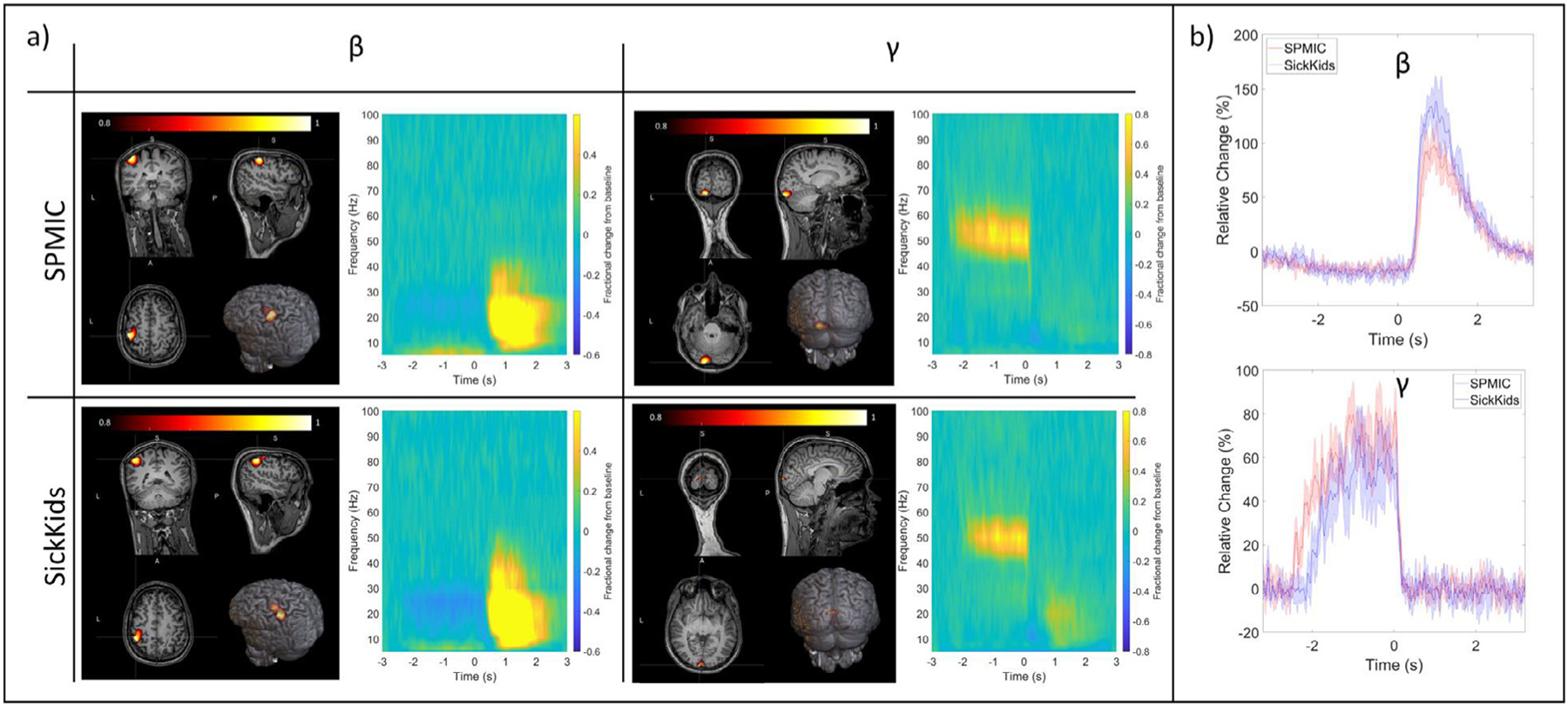
Visuo-motor results (source-level). a) The left and right columns show beta- and gamma-band results respectively. The upper and lower rows show data from the SPMIC and SickKids sites. In each case, a pseudo-t-statistical image, showing the spatial signature of oscillatory modulation (averaged over all 5 runs) is shown on the left, and a time-frequency spectrum for the locations of peak modulation on the right. b) The trial averaged oscillatory envelopes for the beta- (top) and gamma-band (bottom) with SPMIC shown in red, and SickKids in blue with shaded error bars showing the standard deviation across all 5 runs.

Fig. 4 shows the results of source reconstruction for the visuo-motor data. The spatial signature of the change in beta and gamma power can be seen for both systems, as well as time-frequency spectrograms (averaged over runs) and the trial-averaged oscillatory envelopes for the peak location of each band. As expected, the beta modulation maps to the contralateral primary sensorimotor cortex, while the gamma modulation maps to primary visual cortex. For the beta band, the average Euclidean distance between the peaks in sensorimotor cortex for the SickKids and SPMIC data was 12 mm. Within a single site, the peak “scatter ” (calculated as the Euclidean distance from the mean peak location (across 5 runs) to the peak locations identified in each individual run (i.e., a measure of the variability of the peak locations across 5 runs)) was 1.9 ± 0.6 mm for the SPMIC data and 1.7 ± 0.2 mm for the SickKids data. For the gamma peak in visual cortex, the discrepancy across the two sites was 23 mm and the peak scatter was 10.7 ± 2.5 mm for SPMIC and 9.5 ± 7.6 mm for SickKids. Note here that the peak scatter within sites was less than the spatial discrepancy between sites. This could be an effect of co-registration error; recall that all runs at a single site were recorded with the helmet in a single position on the head (i.e., the helmet was not removed between runs), and so a single co-registration of the helmet to the brain anatomy was used. For this reason, co-registration error has no effect on within-site scatter but does affect the between site discrepancy. An additional finding was that the discrepancy and scatter were larger for the gamma band than for the beta band. This will be addressed further below.

The source reconstructed time-frequency spectrograms and trial-averaged oscillatory envelopes also show the same characteristic patterns as the sensor-level data with the movement-related beta decrease and post movement rebound, and visual induced gamma amplitude increase both clearly visible; specifically, the temporal correlations between the trial averaged oscillatory envelopes were 0.95 ± 0.01 in the beta band and 0.81 ± 0.05 in the gamma band. The beta-band responses have SNR values of 47 and 48 for SPMIC and SickKids respectively; the gamma-band values are 16 and 8.

### 3.4. Face processing task results

**Fig. 5.**
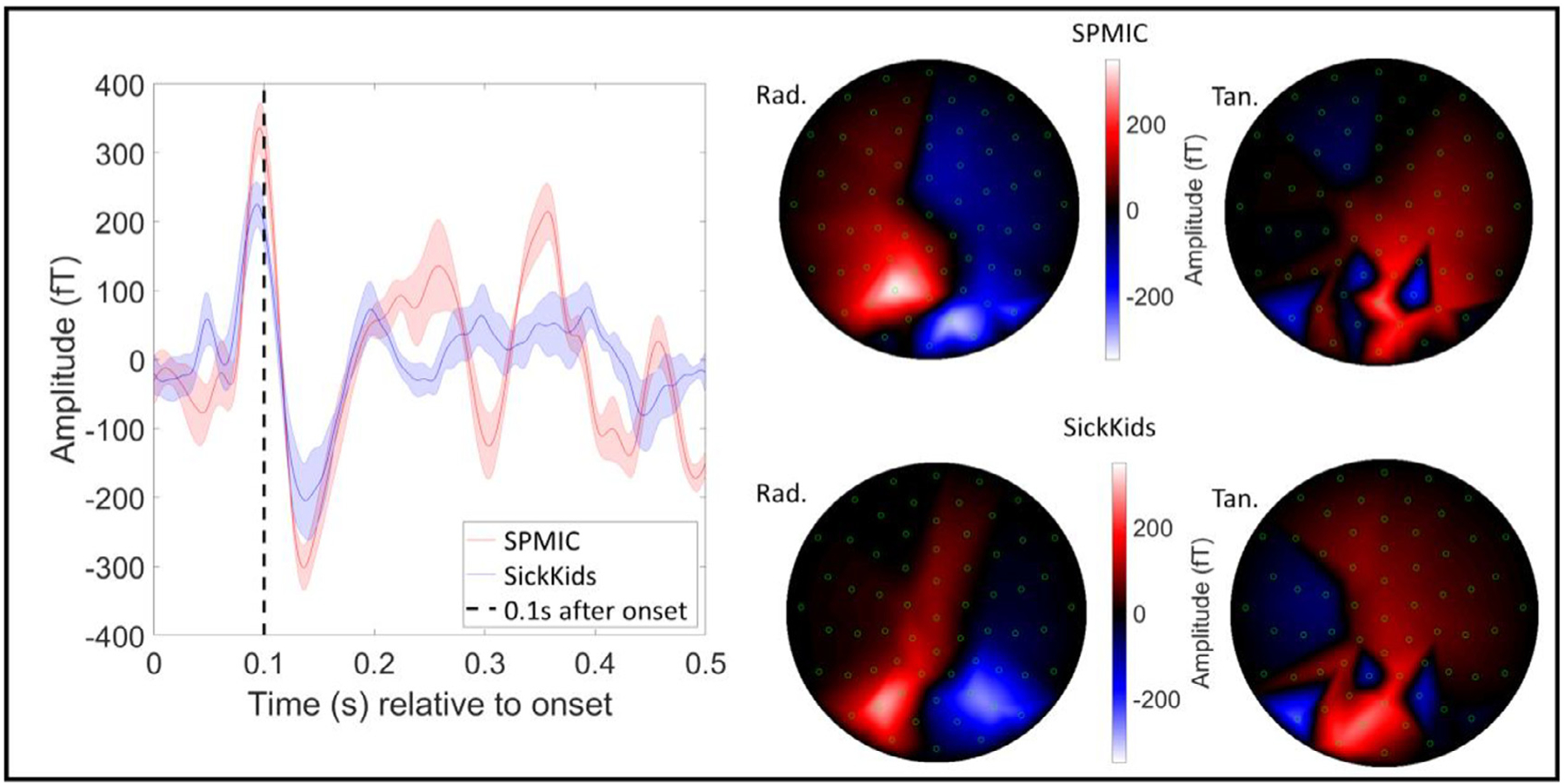
Face processing results (sensor-level). The trial-averaged response in the best sensor for all 5 runs at each site averaged over runs, with the standard deviation across runs shown by the shaded error bars. The dashed line shows 0.1 s after stimulus onset. The best sensor was determined by the range of the trial-averaged signal for each sensor in the 0.075 *s* < *t* < 0.175 s window. The field maps on the right show the field distribution at the peak of the average evoked response at 0.1 s (Z-axis (radial) measurements on the left, Y-axis (tangential) on the right).

Fig. 5 shows a comparison of the evoked responses measured at the sensor level at both sites during the face processing task. The left-hand figure shows the evoked response from the single “best ” sensor; SPMIC data shown in red and SickKids data shown in blue. In both cases, data are averaged across all five runs with the shaded area showing the standard deviation across runs. Both sites show similar responses with a peak in field occurring at ~100 ms post stimulation. The field maps on the right of the Figure show the spatial topography of magnetic field across sensors at 100 ms after onset. The two maps show a similar field distribution with a clear dipolar pattern across the occipital sensors in the Z (radial) components. Note the variation between sites is likely because the helmet was positioned differently on the participant’s head.

**Fig. 6.**
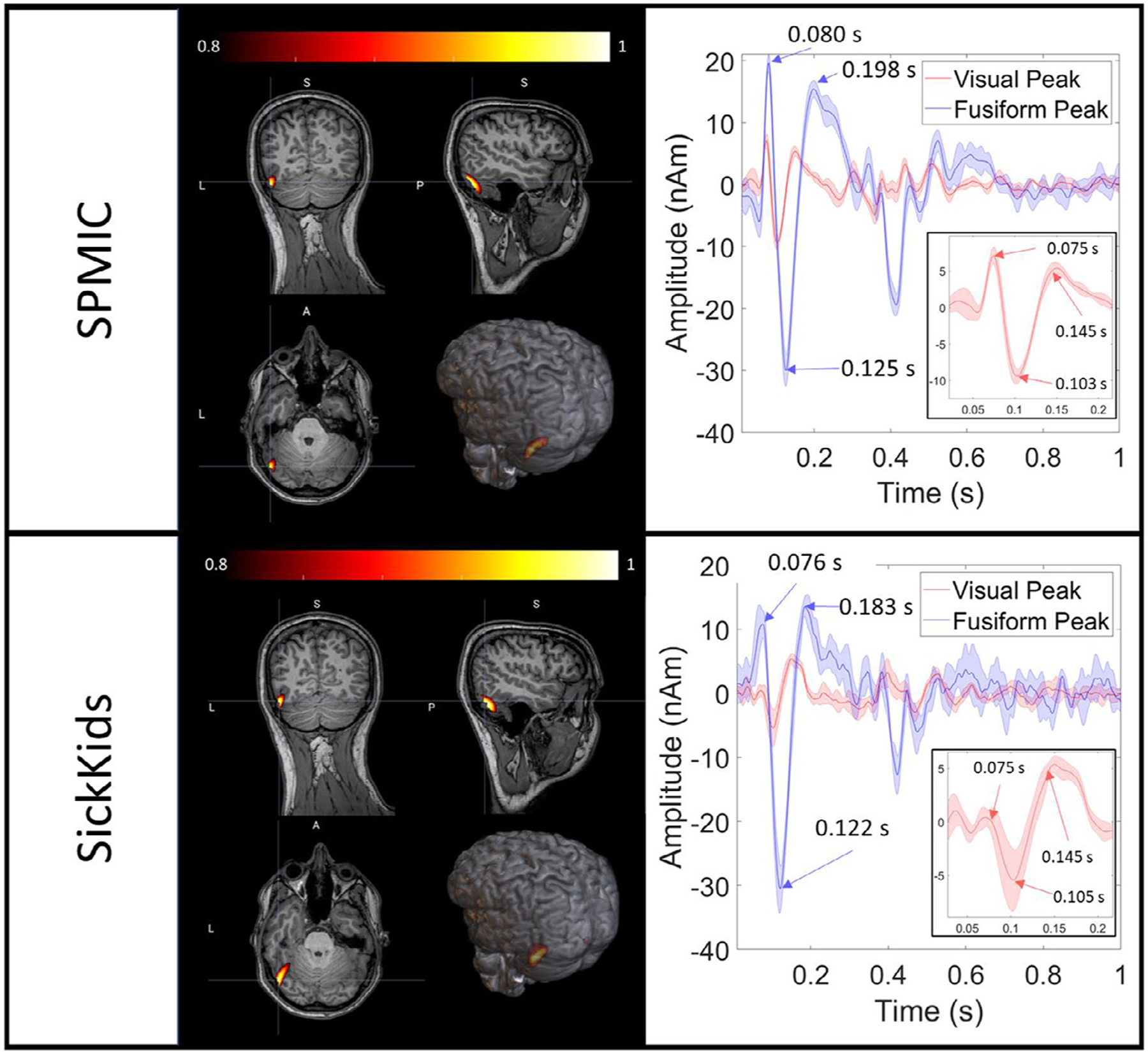
Face processing results (source-level). For each site, the spatial signature of evoked power in the 0.075 *s* < *t* < 0.175 s window is shown contrasted against power in the 1.075 *s* < *t* < 1.175 s window. On the right, two trial-averaged evoked responses are displayed with the shaded error bars showing the standard deviation across runs. The red line corresponds to a time-course reconstructed in the primary visual area, and the blue line to the left fusiform. Note the differences between regions, but also the similarities across sites. Inset, the primary visual response is magnified.

Finally, Fig. 6 shows the source localisation and reconstruction of the evoked response. The peak in the pseudo-t-statistical image is found at the border of the left temporal and occipital lobes for both sites (MNI coordinates: (−46, −68, −11) mm and (−46, −64, −8) mm for SPMIC and SickKids respectively). Two virtual sensor traces are also shown, one extracted from primary visual cortex, and the second from the left fusiform areas for each site (represented as red and blue lines respectively). Inset, the primary visual response is magnified to show the characteristic 75 ms and 145 ms latency peaks. Data from both sites are highly comparable. Spatially, the peak locations in the temporal lobe are separated by 5.7 mm. In terms of temporal morphologies, the two sites are also very similar with near identical latencies for the peak responses, and comparable amplitudes at both virtual sensor locations. Quantitatively, the temporal correlations between the trial averaged evoked responses (measured in the 0 s to 0.3 s window) were 0.70 ± 0.12 in the primary visual area and 0.88 ± 0.04 in the fusiform area. These again show the strong similarity of measured responses across the two sites.

## 4. Discussion

In this paper, we have shown the first cross-site OPM-MEG comparison. Two near identical systems were constructed in different magnetic environments, with a ~25-fold difference in static background field and a ~30-fold difference in low frequency drift. We showed that, through a combination of background field control (dynamic nulling) and post-processing techniques (homogeneous field correction), OPMs not only remain operational in the non-optimised magnetic environment, but also demonstrate a noise floor of ~16 fT/sqrt(Hz) which is only slightly higher than our low noise environment (~10 fT/sqrt(Hz)). In our human experiments, with the same participant scanned multiple times, we were able to record robust, high-quality MEG data in both environments. Specifically, we were able to reconstruct both beta- and gamma-band modulation in our visuo-motor task, and evoked responses in our face processing paradigm. On average, the spatial discrepancy between localisations at the two sites was of order 10 mm. The temporal correlation between the sites was 0.82 ± 0.06 (collapsed across runs and tasks). Thus, both sites showed highly comparable signals, demonstrating that OPM-MEG systems can work reliably, even in busy city centre sites.

Low frequency drift in background field is a significant issue for OPMs, since a shift away from zero has a marked effect on sensor gain. Specifically, a change in field of 3.5 nT causes a change in gain of ~5%; the levels of drift observed in the MSR at the SickKids site were of order 10 nT, which would correspond to ~30% gain change. Without dynamic nulling and field correction, this would be sufficient to invalidate the models used for source reconstruction and render the recorded data an inaccurate representation of brain activity. Previous work has shown that dynamic nulling is effective at cancelling low frequency field drifts (Holmes et al., 2019), although existing demonstrations have been based on lower amplitude artefacts. Here results in Fig. 2 show that low frequency drifts up to 10 nT could be controlled successfully, allowing the sensors to remain within their operational range, measuring high fidelity MEG data. Dynamic nulling is therefore critical to enable successful OPM-MEG operation. However, the precise methodology used has some constraints. First, the reference array used included only 4 sensors at both sites; whilst this enables accurate characterisation of background field close to the helmet, field gradients can only be calculated in a single orientation (Z). Second, the power spectra in Fig. 2 show that dynamic nulling impacts on interference at higher frequencies. This is due to dynamic range: we have to generate fields of order 10 nT, and the dynamic range of the current drivers means a least significant bit size of ~1 pT. Consequently even the smallest changes in current through the coils generate a shift in background field which is large relative to the target noise floor (15 fT/sqrt(Hz)). Thus, any noise is transmitted to the OPMs around the head by the coils themselves, raising the noise floor at all frequencies. Whilst our dynamic nulling scheme worked well, improved reference array design and lower noise current drivers would likely further improve recordings.

Although interference was high following dynamic nulling, it was adequately controlled via the application of homogeneous field correction, which was able to reduce the noise floor at high frequencies (10 Hz – 40 Hz) from ~100 fT/sqrt(Hz) to ~16 fT/sqrt(Hz). HFC is an attractive solution to removal of interference in an OPM array; it is simple to implement, and the low rank of the model (i.e., the assumption of homogeneity across the head sensors) means a low likelihood of removing neural signal. This is especially important in OPM arrays as they typically contain fewer sensors than a SQUID array, hence the likelihood that any spatial basis set will explain the neural signal by chance is increased. However, HFC is extremely dependent on knowledge of the relative orientations of each sensor. For rigid additively manufactured helmets as used in the current study, where the relative sensor locations and orientations are precisely known (from the electronic CAD file used to define the 3D print), this issue is reduced. However, if flexible (EEG-like) caps were used (e.g., as in Hill et al., 2020) the relative sensor locations and orientations are more challenging to measure and could even change throughout an experiment. It is therefore likely that the utility of HFC would decline in this case.

Despite limitations of both the interference rejection methods used, we showed that systems at both sites produced high fidelity data. Temporally, all responses showed good agreement, with beta, gamma, and fusiform evoked responses all exhibiting > 80% correlation. In addition, all responses at both sites were of comparable amplitude. At sensor level, the beta rebound was higher at the SickKids whilst the gamma amplitude was higher at the SPMIC site. Interestingly, in both cases response amplitudes were more comparable following source space modelling. This likely suggests that the effect was due to the placement of the helmet – meaning sensors are either closer to the scalp, or on the scalp surface but closer to the field maxima, for the site with the higher sensor space amplitude. This said, once certainly can’t rule out differences being driven by physiological effects – e.g., the way in which the stimulus was viewed, or the state of the subject, time of day, etc. Of course, whilst e.g., in the gamma band, the effect of signal amplitude all but disappeared when reviewing source space results (i.e., see Fig. 4b), an SNR discrepancy remains.

Spatially, we observed good agreement in source localisation for both the beta band effects in sensorimotor cortex (~10 mm), and the evoked response in left fusiform area (~5 mm). The spatial differences across sites for the gamma band effects in visual cortex were larger (~28 mm). There are several likely reasons for this. Firstly, the stimulus was a centrally presented circular grating, which means the spatial extent of the cortical regions activated will likely be larger than the spatial discrepancy itself. Related, depending on how the screen was set up, and how the subject viewed it, the retinotopic organisation of the visual cortex is likely to result in a spatial shift of the peak in the response (e.g., if it appeared mostly in the left visual field at one site, and the right visual field at the other site, this would likely shift the peak across the longitudinal fissure which could easily account for the discrepancy). Further, gamma oscillations are known to be low amplitude. These effects combined will likely cause the spatial difference in the gamma band to be high, not due to instrumental inefficiency but rather due to the nature of the paradigm. For this reason, the spatial discrepancy of 23 mm – which is high compared to what would be required for e.g., epilepsy surgical evaluation – should not be over-interpreted. Indeed, the equivalent values for the motor response (10 mm), and the fusiform evoked response (5 mm) are closer to what one might expect; in both cases, the precise location and extent of the brain tissue activated less likely to be affected by the stimulus (e.g., it will always be the motortopic representation of index finger; or the face processing area in fusiform gyrus). So, these lower values are more reflective of the true accuracy of the system. An additional point here is that co-registration error is likely to have contributed to the spatial discrepancies between sites. Indeed, when we look at the scatter of peak locations identified within each site, the spatial discrepancy between sites, in beta and gamma peak location, was larger than within site scatter. Future cross site comparisons could avoid this caveat via the use of a bespoke helmet which fits the individual being scanned and removes the need for co-registration (Seymour et al., 2021).

A limitation of the SickKids site remains the static field. The methodology implemented uses a PI controller to dynamically null field drifts that are measured by the reference array; it is this that enables the OPMs to operate at the SickKids site. However, the present implementation still uses on-board sensor coils (rather than the larger bi-planar coils) to null the static (i.e., temporally stationary) field. The currents through these on-board coils are set at the start of an experiment and remain constant throughout. For this reason, any large movements of the head during the scan would mean that the on-board-sensor coil-currents are no longer appropriate for nulling the field at each sensor, and this may cause large magnetic artefacts, or even gain changes (similar to those that we have corrected due to environmental drifts). For this reason, we ensured that for the experimental data shown, whilst the subject was unconstrained, they were asked to remain still during the scan. A better solution would be to use the bi-planar coils to remove both the drift and static field, thus enabling free head movement. However, this is a non-trivial extension since one first has to measure the absolute static offset. It is, in principle, possible to measure static fields using an OPM, however such measurement requires extensive calibration since any material which has been even slightly magnetised, inside the OPM, generates a field offset such that static measurement is compromised. A more attractive method was recently presented and implemented by Rea et al., whereby with dynamic nulling applied, deliberate head movement is used to sample the static field and a fitting algorithm used to determine its magnitude and direction. Implementation of such a method could readily enable free subject head movement in the SickKids environment; this should be a topic of future work.

It should also be noted that, even after dynamic nulling and HFC are applied, sources of interference remain at the SickKids site due to the busy clinical environment and city centre infrastructure in which the system is located. Such interference, which is likely due to e.g., vibrations in the MSR (for example, from a car driving underneath) led to a greater number of trials being rejected at SickKids, compared to SPMIC (particularly in the face processing paradigm). Despite this, a simple trial rejection algorithm was able to discard trials with high degrees of interference. In future installations, the use of an OPM-optimised magnetically shielded rooms, with the capability to demagnetise the inner mu-metal walls (using a degaussing system) will likely reduce these effects.

## 5. Conclusion

We have demonstrated that a commercial OPM-MEG system can be sited in a non-optimised shielded room, in a clinical setting, in a major city and yield data comparable to that collected in an optimised site. Through use of dynamic nulling and homogeneous field correction, this system exhibits low noise, and successfully records OPM-MEG data which are well matched to equivalent data collected using existing (“tried and tested ”) OPM-MEG instrumentation. We stress that the system remains nascent technology; further work can be done on artefact rejection, with more sophisticated post-processing techniques being explored. In addition, both OPM systems will benefit from a larger sensor array, which will improve coverage of the brain, and increase the information available to algorithms like HFC and source localisation, which will further improve signal to noise ratio. Despite this, the paper shows that “plug-and-play” OPM-MEG systems now exist, they can be easily sited even in challenging environments, generate high fidelity data, and will provide significant benefit to clinical research groups who can now begin to exploit the high degrees of practicality and lifetime compliance which OPMs afford, to explore clinical questions.

## Figures and Tables

**Table 1:** Trials rejected. The number of trials (out of 100) rejected for each run at each site.

Visuo-motor task	SPMICNumber of bad trials(Out of 100)	SickKidsNumber of bad trials(Out of 100)
Run 1	12	10
Run 2	13	12
Run 3	26	12
Run 4	13	28
Run 5	18	50
Face PROCESSING task		
Run 1	2	4
Run 2	12	29
Run 3	3	27
Run 4	4	22
Run 5	7	20

## Data Availability

All data were acquired by the authors and are available from the authors upon request. As this is a prototype system, the data are not in a suitable format to be freely shared, and would require the help of an author to access. We are currently working on organising the data into BIDS format so that it can be freely available in the very near future. All code was custom developed in-house using MATLAB, and is available from the authors on request for the same reasons as the data.

## Footnotes

1 This linearity problem could in principle be addressed by “closed-loop ” operation, where on-board sensor coils are used in a feedback loop to maintain zero field inside the OPM, however this is not how the majority of OPMs in the literature operate. Of course, such field drifts also decrease overall SNR (which cannot be controlled by closed-loop operation)
